# The Perspective Structure of Visual Space

**DOI:** 10.1177/2041669515613672

**Published:** 2015-10-30

**Authors:** Casper J Erkelens

**Affiliations:** Helmholtz Institute, Utrecht University, CH, Netherlands

**Keywords:** Visual space, perspective space, alley experiments

## Abstract

Luneburg’s model has been the reference for experimental studies of visual space for almost seventy years. His claim for a curved visual space has been a source of inspiration for visual scientists as well as philosophers. The conclusion of many experimental studies has been that Luneburg’s model does not describe visual space in various tasks and conditions. Remarkably, no alternative model has been suggested. The current study explores perspective transformations of Euclidean space as a model for visual space. Computations show that the geometry of perspective spaces is considerably different from that of Euclidean space. Collinearity but not parallelism is preserved in perspective space and angles are not invariant under translation and rotation. Similar relationships have shown to be properties of visual space. Alley experiments performed early in the nineteenth century have been instrumental in hypothesizing curved visual spaces. Alleys were computed in perspective space and compared with reconstructed alleys of Blumenfeld. Parallel alleys were accurately described by perspective geometry. Accurate distance alleys were derived from parallel alleys by adjusting the interstimulus distances according to the size-distance invariance hypothesis. Agreement between computed and experimental alleys and accommodation of experimental results that rejected Luneburg’s model show that perspective space is an appropriate model for how we perceive orientations and angles. The model is also appropriate for perceived distance ratios between stimuli but fails to predict perceived distances.

## Introduction

Visual space is the space we perceive through vision. Euclid suggested that visual space was confined to a cone having the apex in the eye and the base at the limits of vision (Burton, 1945). In the 18th century, Reid proposed that visual space was spherical on the basis of the shape of the eyes (Suppes, 1977). Luneburg (1947) was the first who attempted to establish the geometry of visual space by combining psychophysical data with mathematical analysis (Rosar, 1985). Although both Hillebrand (1902) and Blumenfeld (1913) performed so-called alley experiments, the geometry proposed by Luneburg was based mainly on data of Blumenfeld. Blumenfeld (1913) used two experimental tasks consisting of positioning alleys of small flames by making judgments about their parallelism and equidistance. The claim made by Luneburg was that the different results from the two alley experiments were evidence for a non-Euclidean geometry of visual space. Luneburg (1947, 1950) proposed a hyperbolic geometry possessing negative Gaussian curvature. Blumenfeld’s alley experiments have been repeated and extended several times (Battro, di Pierro Netto & Rozenstraten, 1976; Hardy, Rand, & Rittler, 1951; Indow, Inoue, & Matsushima, 1962; Shipley, 1957; Zajaczkowska, 1956). All studies concluded that visual space is curved although a few authors challenged its hyperbolic nature. Foley (1972) conducted experiments in which subjects judged visual angles, that is, angles between visual directions, and ratios between perceived frontal and egocentric extents. He found that the ratio of perceived frontal to egocentric extent greatly exceeded the physical ratio, while perceived visual angles corresponded closely to physical ones. Foley (1972) concluded that perceived distances and perceived visual angles are the product of different and independent processes. A possible consequence of Foley’s conclusion is that perceived angles and distances do not constitute a single visual space. Later studies did not consider this possibility and persevered in constructing curved visual spaces by combining angles and distances (Battro et al., 1976; Cuijpers, Kappers & Koenderink, 2000, 2001, 2002; Higashiyama, 1984; Indow & Watanabe, 1984a, 1984b; Koenderink, van Doorn, Kappers, Doumen, & Todd, 2008; Koenderink, van Doorn, Kappers, & Todd, 2002; Koenderink, van Doorn, & Lappin, 2000; Koenderink, van Doorn, & Lappin, 2003; Musatov, 1976; Schoumans, Kappers, & Koenderink, 2000; Todd, Oomes, Koenderink, & Kappers, 2001; Wagner, 1985). All authors compared their experimental results with predictions of Luneburg’s model of a curved visual space and reported all sorts of deviations. Dependence of results on task, reference, distance, and instruction questioned Luneburg’s model of visual space and culminated in even questioning visual space itself (Koenderink et al., 2008). Here the effort is undertaken to investigate whether a model of visual space that is very different from Luneburg’s model can restore the entity of visual space.

Linear perspective used by artists starting from the Renaissance is a mathematical system for projecting three-dimensional scenes onto two-dimensional surfaces, such as paper, canvas, or a screen. Parallel lines receding in the three-dimensional scenes converge to a vanishing point in such projections. Furthermore, distant objects are imaged smaller than equally sized near ones. Linear perspective prescribes how three-dimensional scenes are imaged on surfaces. It does not prescribe how we perceive such scenes in pictures and in the free field. From just looking at a straight railway line or road, it is obvious that perspective is present in visual perception of real three-dimensional scenes and, thus, a property of visual space. Actually, it is odd that visual space is such a deformed representation of physical space. In view of plasticity of cortical maps (Singer, 1995), one would expect that visual space would adapt to long-term and systematic deviations from physical space. Apparently, adaptation does not occur. Instead, human beings have both perspective and Euclidean representations of physical space at their disposal. For example, we see on the one hand that a road narrows in front of us but on the other hand we are confident that it does not. The availability of different representations gives human beings the possibility to answer questions about spatial relationships in different ways. For instance: What is the angle between rails of a railway track or between the lane dividers of a road? One answer expresses properties of visual space whereas the other may express experience with physical space in general and knowledge of rails and roads in particular. Recently, I argued that we see the world in perspective not because we view it from one or two vantage points, but because lines vanish at a finite distance (Erkelens, 2015a).

Perspective has not been considered as a constituent of models of visual space, probably because experiments have usually been conducted in near space where influence of perspective is assumed negligible. However, this argument does not justify the complete absence of perspective in discussions of visual space. For instance, Battro et al. (1976) used distances up to 240 m in their alley experiments without even mentioning perspective in their paper. Recent studies showed that angles perceived between lane dividers of a straight road (Osa et al., 2011) or between rails of a straight railway track (Erkelens, 2015a) were remarkably large. Depending on eye height, perceived angles ranged between 20° and 70° in individual subjects. In a curved visual space, such large perspective angles, distinctive of a highly curved space, are incompatible with perceived distances of vanishing points of many hundreds of metres. The purpose of this study is to explore the geometry of spaces that are perspective transformations of the Euclidean, physical space. Another purpose is to investigate whether such a space, dubbed perspective space, can explain the experimental results of previous studies of visual space, among which the classic parallel and distance alley experiments of Blumenfeld (1913).

## Geometry of Perspective Space

Visual space as a perspective transformation of Euclidean space has hardly been explored in the literature (Lehar, 2003). This is curious because perspective is so obviously a property of visual perception, especially at long distances. Vanishing points at a specific finite distance are a distinctive property of perspective space (Erkelens, 2015a, 2015b). Both physical space and retinal images can be regarded as limiting cases of perspective space because physical space is characterized by vanishing points at infinite distance and retinal images are characterized by vanishing points at zero distance. In this respect, perspective space constitutes a natural bridge between the geometric properties of retinal images and physical environment.

**Figure 1.**
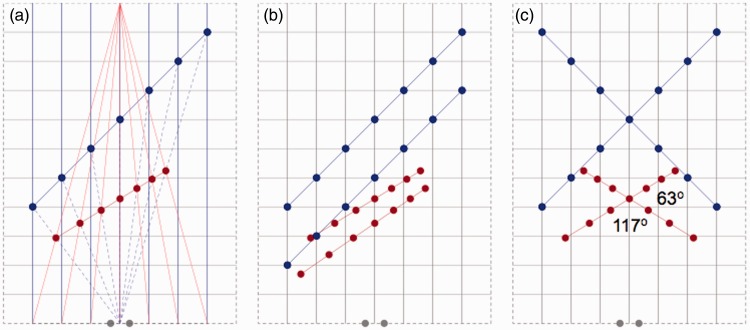
Transformations from physical to perspective space. Panels show stimuli (blue dots and lines) in a plane of physical space and their equivalents in perspective space (red dots and lines). Gray dots indicate the positions of the eyes. Panel (a) shows a set of seven dots (blue) arranged along a straight line in physical space. The dots are lying on equidistant lines (blue) that vanish at infinity in the straight-ahead viewing direction. In perspective space the lines (red) converge to a finite vanishing point. Dots have identical egocentric directions in physical and perspective space (dashed blue lines). Panel (b) shows dots (blue) arranged along two parallel lines in physical space and their equivalents (red) in perspective space. Panel (c) shows dots (blue) arranged along two orthogonal lines in physical space and their non-orthogonal equivalents (red) in perspective space. For reasons of clarity the underlying directional and perspective lines are not drawn in panels (b) and (c).

To explore the geometry of perspective spaces, computations were made on points and lines in physical space. Figure 1a shows the basic idea behind the computations. The Cartesian grid represents a plane in physical space that includes the centers of both eyes. The blue lines represent parallel and equidistant lines in the straight-ahead viewing direction in physical space. Lines converging to a vanishing point at finite distance in perspective space, which are associated with lines in parallel to the viewing direction in physical space, are dubbed perspective lines from now on. The red lines in Figure 1(a) are perspective lines associated with viewing in the straight-ahead direction. Each position in physical space is specified by an egocentric direction and distance. Using a polar coordinate system is appropriate for vision because directions and distances result from different processes. Information about directions follows from retinal and eye position signals whereas distances require interpretation of nonpositional properties of the retinal stimulus (Erkelens, 2012). In the computations we assume that directions of objects in perspective space are identical to their egocentric directions in physical space. The position of a stimulus in perspective space is computed from its position in physical space by finding the intersections between directional and perspective lines. In this way, the distances of stimuli are given by the structure of perspective space. The distance of the vanishing point determines the distance of stimuli in perspective space. The distance of the vanishing point represents the weighted sum of all depth cues. If depth cues such as disparity, size, blur, and ocular vergence together would indicate veridical depth, then the vanishing point would be located at infinity. Depth is not veridical in perspective space. The distance of the vanishing point indicates the underestimation of depth. Figure 1(a) shows that a straight line-piece in physical space (the row of blue lines) transfers to a straight line-piece in perspective space (the row of red dots and lines). Conserved straightness implies that collinearity is preserved in perspective space. Figure 1(b) shows that two parallel line-pieces in physical space are generally not parallel in perspective space. Fronto-parallel line-pieces are the exception. Implication of the directional differences is that parallelism is not preserved in perspective space. Both properties, that is, violated parallelism and preserved collinearity, are also properties of visual space. Evidence comes from experiments in which subjects were asked to set bars in parallel (Cuijpers et al., 2000) and collinear (Cuijpers et al., 2002). Figure 1(c) shows that angles between orthogonal lines in physical space are non-orthogonal in perspective space. Similar differences between physical and perspective angles were measured in a recent study in which subjects judged angles between bars oriented in depth (Erkelens, 2015b). Agreement between computations and experimental results regarding preserved collinearity, violated parallelism, and large angular deviations shows that perspective space is an attractive candidate model of visual space as far as directions and angles are concerned.

**Figure 2.**
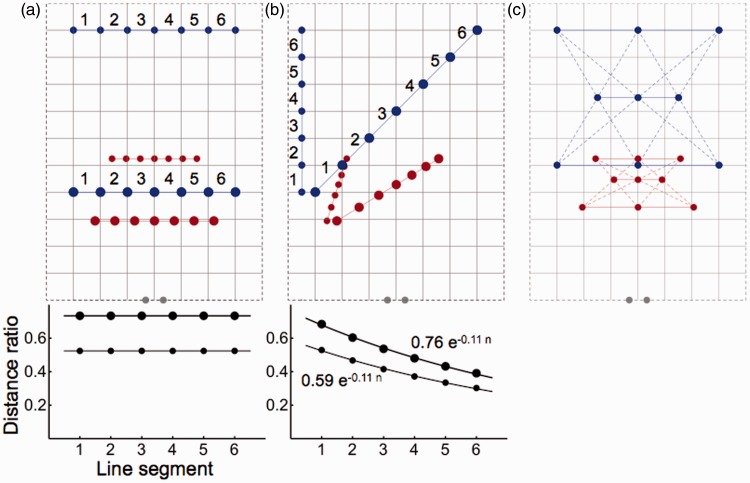
Transformation of distances from physical to perspective space. Panels show stimuli (blue dots and lines) in physical space and their equivalents in perspective space (red dots and lines). Gray dots indicate the positions of the eyes. Panel (a) shows two sets of blue dots in a plane in physical space arranged along straight, frontal line-pieces at different distances from the viewer (big dots at short distance and small dots at long distance). Their perspective equivalents are shown in red. Line segments are numbered from 1 to 6. Ratios between interdot distances in perspective and physical space are shown at the bottom. The horizontal lines through the data indicate that ratios are constant for dots placed along a frontal line. Panel (b) shows similar sets of dots and line segments arranged along other orientations. Ratios between distances in perspective and physical space are shown at the bottom. Lines are best exponential fits to the data. Panel (c) shows the Pappus condition in physical (blue) and perspective (red) space.

Figure 2 shows relations between distances and distances in physical and perspective space. Due to the finite vanishing point in perspective space, distances are shorter than their equivalents in physical space. Distances in perspective space decrease relative to distances in physical space with increasing egocentric distance. The graph at the bottom of Figure 2(a) illustrates the relationship by showing the highest ratio between distances in perspective and physical space at the shortest egocentric distance. Figure 2(b) shows the relationships for sizes of differently oriented line-pieces. The graph at the bottom indicates that ratios between perspective and physical sizes decrease approximately exponentially as a function of distance in the depth direction. Figure 2(a) and (b) shows that sizes are not invariant under translation and rotation in perspective space. The size ratios are consistent with experimental evidence that perceived sizes are underestimated (Gilinsky, 1951) and compression of size in depth relative to frontal size increases with distance (Li, Phillips, & Durgin, 2011). Perspective space as a model of visual space provides geometrical explanations for these experimental findings.

Experimentally, Koenderink et al. (2002) found that visual space fulfills the Pappus condition, implying that visual space is a homogenous projective space. The Pappus condition is fulfilled if connections (dashed lines in Figure 2(c)) between two sets of collinear triples of points (the top and bottom rows of blue and red dots) constitute a collinear triple of intersections (the middle rows of blue and red dots). Positions of the set of red dots in Figure 2(c) were computed by transforming the positions of dots from physical to perspective space. The middle row of red dots shows that the intersections are collinear in perspective space. The Pappus condition is fulfilled because collinearity is preserved in perspective space (Figure 1(a)).

## Dependence on Vanishing Distance and Fixation Direction

**Figure 3.**
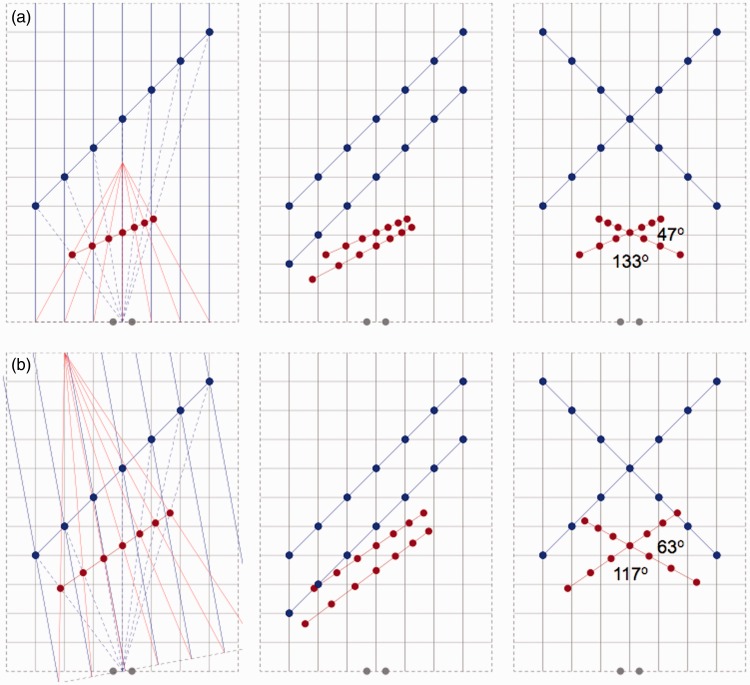
Effect of vanishing distance and fixation direction on perspective space. Panels show physical stimuli (blue dots and lines) and their equivalents in perspective space (red dots and lines). Gray dots indicate the positions of the eyes. Panels (a) show the panels of Figure 1 but now for the conditions that the vanishing point is at half the distance. Panels (b) show the panels of Figure 1 for a fixation direction of about 10° to the left side.

Perspective space as introduced in the previous section contains two parameters, that is, distance of vanishing point and fixation direction. It is obvious that the parameters may vary across observers and conditions. Distance of vanishing point may depend on context as was recently shown in judgments of perspective angles between rails (Erkelens, 2015a). Distances were about 5 m for judgments made in a full-cue, natural environment and about 0.3 m for judgments made from pictures of the same scene. Fixation direction is fixed in some psychophysical experiments but not in others and certainly not under natural viewing conditions. It is relevant to know how the two parameters affect the positions of objects in perspective space. Figure 3 shows the positions of points in perspective space for a short distance of the vanishing point (Figure 3(a)) and an oblique direction of fixation (Figure 3(b)).

Comparison of Figures 1 and 3 shows that different vanishing distances and fixation directions do not affect perspective space in qualitative terms. The main properties, that is, preserved collinearity, violated parallelism, and angular deviations, are retained in the different conditions. Quantitatively, however, there are differences. Distance of vanishing point has a substantial effect on orientations of line-pieces in perspective space (Figure 3(a)). A shorter distance of the vanishing point (by a factor of 2) was associated with larger perspective angles (133° vs. 117°). Similar results were found for judgments made in the natural environment and from pictures of perspective angles of roads and rails (Erkelens, 2015a; Osa et al., 2011) and perspective angles between converging and diverging bars (Erkelens, 2015b). It seems a fair interpretation of the experimental findings that contextual differences affect vanishing distance and perspective angles. Based on other types of experimental results, other authors have earlier argued that the geometry of visual space depends on contextual conditions (Foley, 1972; Schoumans et al., 2000; Suppes, 1977). The oblique direction of fixation slightly affected the orientations of line-pieces in perspective space. The subtle differences are visible in Figures 1 and 3 by closely comparing the positions of dots and lines. The change in fixation direction of 10° to the left resulted in a leftward rotation of 4° of all line-pieces. As a consequence, perspective angles between line-pieces were preserved. Small but significant effects of fixation have been reported for measurements of the geometry of visual space (Ehrenstein, 1977) and for bisection judgments in visual grasp space (Trommershäuser, Maloney, & Landy, 2003).

## Blumenfeld’s Parallel and Distance Alleys

**Figure 4.**
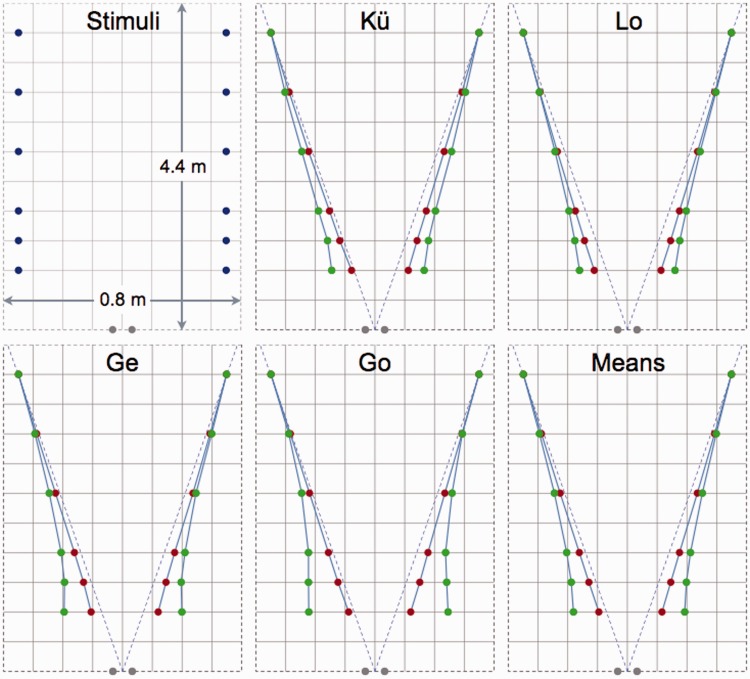
Parallel and distance alleys as measured by Blumenfeld (1913)**.** Panels show the stimuli at their initial locations (blue dots) and the mean settings of four subjects under the instructions of parallelism (red dots) and equidistance (green dots). The panel labeled *Means* shows the settings averaged across the four subjects. Gray dots indicate the positions of the eyes.

A critical test for the perspective space model is being able to describe the parallel and distance alleys of Blumenfeld (1913) because on the basis of these alleys Luneburg (1947, 1950) concluded that visual space had to be a Riemannian space of constant curvature. To make the alley results accessible to computation, Blumenfeld’s data were reanalyzed and rearranged. Blumenfeld (1913) reported the parallel and distance alleys in an extensive paper of 160 pages including 73 tables, written in the German language. Each table lists the settings made by one subject under one (parallel or equidistant) instruction. Four subjects produced complete sets of data in both types of alley experiments. Blumenfeld (1913) did not provide an oversight of these data. Therefore, parallel and distance alleys were constructed from Blumenfeld’s tables for these four subjects (Figure 4). Mean settings were computed of all settings made by individual subjects. No distinction was made between measurements in which stimuli were presented simultaneously or in succession.

Stimuli and results are presented here in the format used by Blumenfeld (1913), which means that frontal distance is amplified by a factor of four relative to distance in the depth direction. The square elements of the grids shown in Figure 4 represent frontal distances of 0.1 m and in-depth distances of 0.4 m. Amplification of frontal distances, which has also been applied by later presenters of alleys (Angell, 1974; Battro et al., 1976; Blank, 1953; Hardy et al., 1951; Indow et al., 1962; Luneburg, 1950; Roberts & Suppes, 1967; Shipley, 1957; Yamazaki, 1987; Zage, 1980; Zajaczkowska, 1956), visually emphasizes, and thus exaggerates, the differences between parallel and distance alleys. Stimuli were little flames at the end of thin vertical tubes in a further dark room or thin vertical rods in a lit room. The flames (rods) could move only sideways except for the two flames (rods) at the far end, which stood at fixed places. Subjects received very detailed instructions. For the parallel-alley experiment, the instructions were as follows: “Adjust the little flames (rods) so that the (virtual) lines connecting them with the most distant flames (rods) appear to you as parallel, symmetrical, straight lines, that is, that the two rows from front to rear and back do not converge or diverge. The observation must be made with free viewing along the rows, if possible, in a casual manner”. For the distance alleys, the instructions were as follows: “Adjust the lights so that the lateral distance between the two flames of each pair appears equal to that of the farthest pair. It is not necessary that the interflame distances of the other pairs seem equal to each other. If possible, one should not look at the rows oriented in depth on each side. It is not allowed to use the absolute size of a distance expressed in any objective measure (cm or m) during the comparison of distances”. Figure 4 shows that the subjects produced rather symmetric alleys, with little intersubject variability. All subjects set the distance alley wider than the parallel alley. The flames (rods) were placed in almost straight lines in the parallel-alley experiment. The trajectories were curved outward in the distance-alley experiment mainly as a result of the settings of the near pairs of stimuli.

## Computation of Blumenfeld’s Alleys

**Figure 5.**
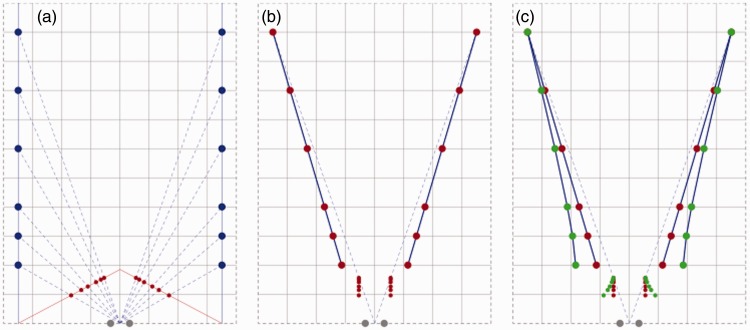
Computed parallel and distance alleys. Panel (a) shows Blumenfeld’s stimuli (blue dots) arranged along two parallel and equidistant lines (blue lines). Blue dashed lines indicate their egocentric directions. In perspective space the stimuli (small red dots) are aligned along two (red) lines converging to a vanishing point located at a finite distance from the observer. Panel (b) shows how the stimuli in physical space (red dots connected by blue lines) have to be positioned in order to align the stimuli in two parallel rows in perspective space (small red dots). Panel (c) shows the computed parallel (red dots) and equidistance (green dots) alleys in physical space together with the parallel alleys (small red dots) and equidistant alleys (small green dots) in perspective space.

Blumenfeld’s parallel and distance alleys were computed by transforming positions of stimuli from physical space to perspective space and vice versa. First, the settings were computed for the parallel-alley task. To that end positions in perspective space were computed for Blumenfeld’s stimuli at their initial positions in physical space (Figure 5(a)). Stimuli (red dots) had egocentric directions in perspective space that were identical to those in physical space but were lying on two perspective lines meeting at a certain finite vanishing point. At this point of the computation, the choice for the position of the vanishing point was provisional because its final location depended on the final settings of the stimuli in physical space in the parallel-alley task. The next operation was to shift the stimuli sideways in perspective space so that they formed a parallel alley (Figure 5(b)). The shifts were made sideways because in Blumenfeld’s experiments the stimuli could only be displaced sideways in physical space. In perspective space, the farthest stimulus was kept at a fixed position because in Blumenfeld’s experiments the farthest stimulus had a fixed position in physical space. The positions of the stimuli in physical space were computed from the new positions of the stimuli in perspective space by finding the intersections between the new directional lines and the frontal lines along which the stimuli could move in physical space. Finally, the position of the vanishing point was found by trial and error, guided by the criterion that computed settings (Figure 5(b)) were as close as possible to the mean settings of subjects in Blumenfeld’s experiment (Figure 4). A vanishing point located at 0.74 m from the viewer gave the best result. Then the mean deviation of the computed positions was as small as 5.4 ± 5.7 mm for the parallel alley.

After having made their final settings in the parallel-alley task of Blumenfeld’s experiment, subjects reported that the nearer stimuli pairs were set closer to each other than the farthest pair (Blumenfeld, 1913). In the distance-alley task, Blumenfeld asked subjects to adjust the distance between pairs of stimuli so that distances between stimuli were judged equidistant. Consequently, subjects set the distance between nearer stimuli pairs wider in the distance-alley task than in the parallel-alley task. In the current computations, the frontal distance between each pair in the distance alley was obtained by multiplying frontal interstimulus distances of the parallel alley with a factor related to the in-depth distances of the stimulus pairs in perspective space. The distance alley (Figure 5(c)) was computed by choosing the factor of multiplication equal to the ratio between the in-depth distances of farthest stimulus pair and stimulus pair under evaluation. The multiplication factor resulted in very good agreement of the computed distance alley with the experimental settings of Blumenfeld (1913). The mean deviation of the computed positions of the distance alley was just 4.6 ± 3.5 mm.

## Discussion

### Perspective Space as a Model of Visual Space

Perspective is a so familiar property of visual perception that it seems totally unremarkable for both laymen and scholars. Only very recently perspective space was considered as a model of visual space (Erkelens, 2015a, 2015b). Although replacing infinite vanishing points in Euclidean space by finite vanishing points in perspective space may seem a minor operation, the geometry of perspective space is considerably different from that of Euclidean space. Angles and distances are not invariant under translation and rotation as they are in Euclidean space. A common property of both spaces is that they are flat. Properties of perspective space have been investigated here in two dimensions. However, inclusion of the eyes was the only restriction applied to the computations made for line-pieces in two-dimensional planes. Rotation of the planes about the interocular axis generalizes the observed properties of perspective space from two to three dimensions.

Agreement between computational and experimental results shows that perspective space is an appropriate model for visual space, particularly in relation to perceived directions and angles. It is noteworthy that in the perspective space model distance of the vanishing point determines the distances of stimuli. Experimental findings show that perceived distance in the depth direction does not fit well within the concept of perspective space. The computations presented in Figure 2 show that distance in perspective space has an approximately exponential relationship to distance in physical space. Experimentally, very different relationships have been reported, showing just small differences between perceived and physical distances at least up to 20 m (Epstein, 1963; Gilinsky, 1951; Sinai, Ooi, & He, 1998; Wagner, 2006). Furthermore, distances of vanishing points computed from perspective angles were found to be highly incongruent with perceived distances of vanishing points (Erkelens, 2015a). Perspective angles predicted that vanishing points would be as close as just a few metres from the observer. In contrast, distance judgments of the vanishing points gave estimates of many hundreds of metres. Remarkably, the distance alleys of Blumenfeld (1913) were well described by the perspective space model in combination with the size-distance invariance hypothesis. The good and bad fits seem to imply that perspective space is an appropriate model for perceived distance ratios of stimuli but not for perceived distances. This combination of predicted distance ratios and non-predicted distances suggests the existence of a factor that is needed to convert perceived distance ratios into perceived distances. Cognition of distances resulting from experience with the physical world may be the source of this factor.

### Interpretation of Blumenfeld’s Alleys

The parallel alleys in physical space of Blumenfeld (1913) were obtained by computing the physical positions of stimuli associated with parallel alleys in perspective space. Agreement between computed and experimental alleys was very good. The computed alleys were symmetric because a single vanishing point was assumed for both rows of the alleys. Agreement would even have been better if the computational results would have been optimized for stimuli on the left and right rows separately. The perspective model of visual space contains two free parameters, namely, distance and direction of the vanishing point, the former related to context and the latter to direction of fixation. Optimal distance of the vanishing point of 0.74 m may seem extremely short. Judgments of perspective angles of physical rails showed that distance of the vanishing point decreased with eye height and became as short as 1 to 2 m for an eye height of 0.40 m (Erkelens, 2015a). In Blumenfeld’s experiments, subjects viewed the stimuli at eye level or at an eye height of just 0.07 m. Thus, in relation to the railway track experiments, the vanishing distance of 0.74 m is not an unrealistic distance.

The distance alleys were computed from the parallel alleys by multiplying the interstimulus separations with factors related to distance in the depth direction. Again, agreement between computed and experimental alleys was very good. For explaining the distance alleys, it may be relevant to look at the instructions that were given to the subjects in Blumenfeld’s experiments (see Blumenfeld’s Parallel and Distance Alleys). Wagner (2006) described four types of instructions, namely, objective, perspective, apparent, and projective. The objective and perspective instructions ask subjects to adjust the positions and directions of stimuli as they are in physical space. Blumenfeld’s instructions were different. The extensive instructions of Blumenfeld (1913) even assigned subjects to ignore certain properties of the stimuli in physical space. Blumenfeld gave the apparent instructions, which means that he emphasized the subjective or phenomenal experience of parallelism and distance (Wagner, 2006). The projective instruction asks subjects to adjust the angular extent of stimuli. Blumenfeld (1913) did certainly not give this type of instruction in the distance-alley task because subjects should not look at the rows oriented in depth. According to the size-distance invariance hypothesis (Epstein, 1963; Wagner, 2006), the perceived distances between pairs of stimuli were equally large when the stimuli were seen to converge to the finite vanishing point (Figure 5(a)). The consequence of displacing the nearer stimuli inwards such that they became parallel in perspective space (Figure 5(b)) was that perceived distances between nearer pairs of stimuli became shorter. Apparently, equidistance was reestablished by multiplying interstimulus distances between nearer pairs with ratios between far and near stimulus depths. Applying the size-distance invariance hypothesis to the parallel-alley data produced a realistic distance alley (Figure 5(c)) provided that the computations used depth values in perspective space.

The parallel and distance alleys of Blumenfeld (1913) are explained within the framework of perspective space if we assume that subjects mixed up the metrics of both visual and physical spaces. We, human beings, seem to have two notions of parallelism, one related to physical space and another to visual space, although it is not quite clear how we define and calibrate parallelism in visual space. Maybe we compensate for the perceived angle between parallel lines in physical space to arrive at parallelism in visual space. Parallelism is a rather troublesome concept outside Euclidean and affine geometries. Parallelism is equivalent with equidistance in Euclidean space. This equivalence is not valid in Riemannian and perspective spaces. In Riemannian models of visual space, parallelism is assumed equivalent with geodesics (Blank, 1953; Luneburg, 1950). However, it is just an assumption. Ironically, the trajectories orthogonal to geodesics are known as geodesic parallels in differential geometry. In visual space parallelism is not equivalent with equidistance because, if it were, parallel and distance alleys would be indistinguishable. The perspective model of visual space explains the parallel alleys. Figure 5(b) shows that parallelism in visual space is associated with divergent parallel alleys in physical space. The divergent parallel alleys are just slightly different from egocentric directions (blue dashed lines in Figure 5(b)). This fact implies that the observation of Koenderink, van Doorn, de Ridder, and Oomes (2010), that visual rays in physical space seem parallel in visual space, is explained by the perspective structure of visual space. The distance alleys are explained by the model in combination with size-distance invariance. Computed distance alleys resembled measured distance alleys if in-depth distances were used as they are in perspective, and thus, visual space. Using physical distances would have resulted in equidistant alleys as they are in physical space.
